# Epidemiology, Survival, and Second Primary Malignancies in T‐Cell Large Granular Lymphocytic Leukemia

**DOI:** 10.1002/jha2.70036

**Published:** 2025-05-06

**Authors:** Arya Mariam Roy, Sawyer Bawek, Richa Parikh, Muhammad Salman Faisal, Paola Ghione

**Affiliations:** ^1^ Division of Medical Oncology, Department of Medicine, The Ohio State University Comprehensive Cancer Center, College of Medicine The Ohio State University Wexner Medical Center Columbus OH USA; ^2^ Roswell Park Comprehensive Cancer Center Buffalo New York USA; ^3^ Department of Hematology/Oncology Karmanos Cancer Institute/Wayne State University Detroit Michigan USA; ^4^ Memorial Sloan Kettering Cancer Center Buffalo New York USA

**Keywords:** epidemiology, hematological malignancies, leukemia, lymphoproliferative disease

## Abstract

**Introduction:**

Large granular lymphocytic leukemia (LGL) is a rare lymphoproliferative disorder, with limited literature available about the epidemiology, survival, and development of secondary primary malignancies (SPMs) in T‐LGL.

**Methods:**

The Surveillance, Epidemiology, and End Results (SEER) 17 registry was used to identify all cases of T‐LGL diagnosed between 2000 and 2019, and patients with primary T‐LGL were analyzed.

**Results:**

Patients with primary T‐LGL were found to have a higher incidence of SPMs compared to the general population, with increased risk for hematological malignancies seen within the first 10 years and solid tumors seen after 10 years of T‐LGL diagnosis.

**Conclusion:**

Patients with primary T‐LGL were found to have a higher incidence of SPMs. Further research is needed to better understand the reason for this increased risk.

**Trial Registration:**

The authors have confirmed clinical trial registration is not needed for this submission

## 1 Introduction

Large granular lymphocytic leukemia (LGL) is a rare heterogenous lymphoproliferative disorder characterized by clonal expansion of large granular lymphocytes [1]. The pathogenesis of LGL remains unclear but it is widely acknowledged that the stimulation of several pathways, including JAK/STAT, FAS/FAS‐L, RAS‐RAF‐1, MEK1‐ERK, PI3K/AKT, NK‐KB, and the sphingolipid rheostat, is implicated in the proliferation of LGLs [2, 3].

T‐cell and natural killer (NK)‐cell LGL leukemia account for 2%–5% of chronic lymphoproliferative diseases across North America and Europe. T‐LGL, while rare, remains frequently underdiagnosed due to its indolent course and nonspecific symptoms [4]. The conclusive diagnosis of LGL necessitates persistent clonal expansion of T‐cell or NK‐cell LGLs, established through cytology, immunophenotype analysis, and repeated clonality evidence [5]. Standard treatment of T‐LGL and NK‐LGL primarily relies on an immunosuppressive backbone (methotrexate, cyclophosphamide, and cyclosporin A) [6]. However, these medications have poor long‐term tolerability (cyclophosphamide and cyclosporin in particular) and can generate further immunosuppression and cytopenias in these patients, highlighting the need for newer options for these patients.

The association between T‐LGL leukemia and B‐cell disorders have been reported in both indolent and aggressive B‐cell non‐Hodgkin lymphomas [7]. In addition, T‐LGL has been associated with coexisting plasma cell disorders [8]. Patients with certain chronic hematological disorders, in particular chronic lymphocytic leukemia, exhibit a higher rate of secondary primary malignancies (SPMs) compared to the general population; however, the incidence, epidemiology, survival, and development of SPMs in LGL are less understood due to its rarity and limitations in associated literature. As the survival of people with LGL improves with therapeutic advances, knowledge of the incident SPMs and cause‐specific mortality will be critical to counsel patients and guide survivorship strategies.

## 2 Methods

We utilized the November 2021 submission of the SEER 17 registry, which covers ∼26.5% of the US population based on the 2020 census and analyzed the data using SEER*Stat version 8.4.0 statistical software. We identified all cases of T‐LGL diagnosed between 2000 and 2019 using the International Classification of Diseases for Oncology edition 3 (ICD‐O‐3) code 9831/3.

The SEER* Stat Frequency and Case Listing sessions were employed to acquire baseline characteristics of cases. Kaplan–Meier method was used for survival analysis. Utilizing the SEER*Stat Multiple Primary‐SIR Session, we computed standardized incidence ratios (SIRs) and absolute excess risk (AER) for SPMs. SIR, or relative risk, is the ratio of the observed number of each SPM to the expected number of that malignancy in the general population. Statistical significance was determined by the exact method. AER is the surplus cancers beyond the anticipated amount per 1000 persons per year, calculated as ([observed count − expected count] × 1000)/person‐years at risk.

Cases were incorporated in SPM analysis if T‐LGL was their initial and exclusive primary malignancy or if T‐LGL was their first primary malignancy among two or more primary malignancies. SPMs diagnosed within 2 months of T‐LGL diagnosis were excluded from SPM analysis to avoid erroneously including malignancies diagnosed concurrently with T‐LGL in our analysis. All T‐LGL malignancies were primary malignancies and patients with other concomitant malignancies were excluded. We studied both solid and hematological malignancies.

## 3 Results

A total of 2297 cases were identified during the study period. The age‐adjusted incidence rate of T‐LGL in the United States was 0.14 cases per 100,000 individuals. The incidence rate was higher in elderly patients (age ≥ 65 years), and the highest incidence was observed in 80–84 years (0.8 cases per 100,000 individuals). Baseline characteristics of the study cohort are outlined in (Table 1). The overall survival (OS) of the entire cohort at 1‐, 3‐, and 5‐year marks was 99.2%, 84.8%, and 80% respectively. The 5‐year OS was 77.2% for males and 82.6% for females. Caucasians had a 5‐year OS of 78% and other ethnicities had a 5‐year OS of 88%.

**TABLE 1:** Baseline characteristics of T‐LGL and SPM.

	T‐LGL	SPM
Total number of cases (*n*)	2297	139
**Sex**		
Male	1204 (52.41%)	71 (51.08%)
Female	1093 (47.58%)	68 (48.92%)
**Race**		
White	1866 (81.24%)	120 (86.33%)
Black	216 (9.40%)	11 (7.91%)
Asian or Pacific Islander	125 (5.44%)	8 (5.76%)
American Indian/Alaska Native	36 (1.57%)	
Unknown	54 (2.35%)	
Median age of diagnosis (years)	67.0	71.0

Abbreviations: SPM, second primary malignancies; T‐LGL, T‐large granular lymphocytic leukemia.

**FIGURE 1:**
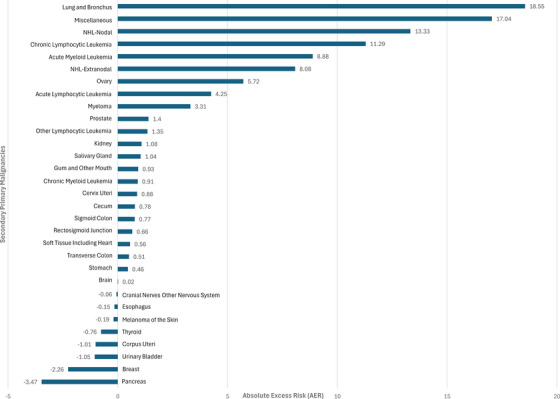
Secondary primary malignancies in T‐LGL. AER, absolute excess risk.

For SIR analysis, 1668 patients with T‐LGL as the primary malignancy were included. A total of 139 (8.3%) patients developed a total of 151 SPM after the diagnosis of T‐LGL. The median latency period for the development of SPMs was 25.5 months (range, 5–209). T‐LGL patients had an overall increased risk of developing SPMs than the general population (SIR 1.48, 95% CI 1.25–1.74, AER 71.45, *p* < 0.05) (Table 2). Among solid tumors, the risk of development of ovarian malignancies (SIR 4.8, 95% CI 1.5–10.7, AER 5.72, *p* < 0.05) was higher in T‐LGL patients, followed by lung and bronchus (SIR 1.88, 95% CI 1.24–2.74, AER 18.55, *p* < 0.05). Acute lymphocytic leukemia (ALL) (SIR 31.33, 95% CI 6.64–91.55, AER 4.25), acute myeloid leukemia (SIR 7.50, 95% CI 3.02–15.46, AER 8.88), chronic lymphocytic leukemia (SIR 7.02, 95% CI 3.21–13.33, AER 11.29), non‐Hodgkin lymphoma, both nodal and extra‐nodal, (SIR 4.35, 95% CI 2.62–6.79, AER 21.41), and miscellaneous cancers which mostly (14/16 cases) included myelodysplastic syndrome (MDS) (SIR 3.67, 95% CI 2.1–5.96, AER 17.04) were the hematological malignancies which had higher risk of occurrence as SPMs in T‐LGL cases (all *p* < 0.05). The overall risk of developing SPM in the T‐LGL group in 1, 1–5, and 10+ years from diagnosis of T‐LGL was significantly higher than in the general population. In 1, 1–5, and 5–10 years from the T‐LGL diagnosis the rate of developing hematological malignancies was higher in the T‐LGL group than in general population (SIR 8.2, 95% CI 4.5–13.8, AER 98; SIR 4.67, 95% CI 2.99–6.94, AER 49.92; SIR 2.84, 95% CI 1.04–6.2, AER 25.2 respectively, all *p* < 0.05). The risk of developing ALL was significantly higher in 1 year after the diagnosis of T‐LGL (SIR 114.66, 95% CI 13.89–414.21, AER 15.81, *p* < 0.05). The overall risk of development of solid tumor malignancies was significantly higher only 10 years after the diagnosis of T‐LGL (SIR 3.11, 95% CI 1.6–5.77, AER 285.11, *p* < 0.05), which is mainly due to salivary gland tumors (SIR 88.33, 95% CI 2.24–492.17, AER 37.75, *p* < 0.05). Overall, tumors of the lung and bronchus (17%), miscellaneous cancers (10.6%), and prostate (9.9%) were the most observed SPMs (Figure 1).

**TABLE 2:** Overall risk of second primary malignancies in patients with T‐LGL.

SPM	Overall SIR (95% CI); excess risk	2–11 months latency SIR (95% CI); excess risk	1–5 years latency SIR (95% CI); excess risk	5–10 years latency SIR (95% CI); excess risk	10+ years latency SIR (95% CI); excess risk
**Overall**	1.48^a^ (1.25–1.74); 71.45	2.17^a^ (1.55–2.95); 171.89	1.31^a^ (1.03–1.65); 45.75	1.14 (0.74–1.67); 20.25	2.67^a^ (1.33–4.77); 262.47
**Solid tumors**					
Ovary	4.58^a^ (1.49–10.69); 5.72	2.17^a^ (1.55–2.95); 171.89			
Lung/bronchus	1.88^a^ (1.24–2.74); 18.55		1.91^a^ (1.07–3.15); 18.89		
Salivary gland					88.33^a^ (2.24–492.17); 37.75
All solid tumors	1.04 (0.83–1.27); 4.47				3.11^a^ (1.55–5.57); 285.11
**Hematological malignancies**					
Acute lymphocytic leukemia	31.33^a^ (6.46–91.55); 4.25	114.66^a^ (13.89–414.21); 15.81			
Acute myeloid leukemia	7.50^a^ (3.02–15.46); 8.88	11.86^a^ (1.44–42.83); 14.61	5.84 (1.21–17.08); 6.58	9.44^a^ (1.14–34.09); 11.61	
Chronic lymphocytic leukemia	7.02^a^ (3.21–13.33); 11.29	12.67^a^ (2.61–37.03); 22.04	7.08 (2.3–16.52); 11.37		
NHL‐extranodal	4.73^a^ (1.9–9.75); 8.08	11.12^a^ (2.29–32.5); 21.78			
All lymphatic and hematopoietic diseases	4.71^a^ (3.42–6.32); 50.7	8.23^a^ (4.15–13.81); 98.1	4.67^a^ (2.99–6.94); 49.92	2.84^a^ (1.04–6.17); 25.21	
NHL	4.35^a^ (2.62–6.79); 21.41	8.79^a^ (3.53–18.11); 49.48	3.75^a^ (1.71–7.12); 17.47		
NHL‐nodal	4.15^a^ (2.15–7.25); 13.33	7.60^a^ (2.07–19.45); 27.71	3.78^a^ (1.39–8.23); 11.68		
Miscellaneous malignancies	3.67^a^ (2.1–5.96); 17.04				

Abbreviations: CI, confidence interval; NHL, non‐Hodgkin's lymphoma; SIR, standardized incidence ratio; SPM, second primary malignancies; T‐LGL, T‐large granular lymphocytic leukemia.

^a^ Denotes statistically significant values, *p* < 0.005; statistical significance was determined by the exact method.

## 4 Discussion

Our comprehensive retrospective study, which is the first study in our knowledge assessing the incidence of SPMs in T‐LGL, provides novel insights into the epidemiology and OS of T‐LGL. The risk of SPM is higher in patients with T‐LGL compared to the general population especially 1, 1–5, and 10+ years from the diagnosis. The increased risk of SPMs in T‐LGL patients was mainly due to a higher number of hematological malignancies seen within the first 10 years and solid tumors seen after 10 years of T‐LGL diagnosis (predominantly due to salivary gland tumors).

We observed that ALL had the highest risk of occurrence among hematological malignancies as a SPM in T‐LGL. Lung and bronchus, ovarian, miscellaneous (mainly MDS), ALL, AML, CLL, and non‐Hodgkin lymphoma were the most common SPMs seen. The enhanced risk of malignancy in the T‐LGL patient population is likely multifactorial and in part due to immunological disorders, exposure to immunosuppressive drugs, chronic inflammation associated with T‐LGL, and possibly due to shared molecular pathways [9]. Interestingly, some common cancers like breast cancer are not overrepresented among LGL patients. Further research is needed to explore this association.

Our study has important clinical implications as it helps emphasize the importance of screening and vigilant surveillance for patients with T‐LGL even beyond 10 years after diagnosis due to the increased risk of developing SPMs. Our study not only unveils new avenues for understanding the heightened risk of SPMs in T‐LGL but also offers potential insights into developing treatment options and prevention strategies for these SPMs. Exploring the impact of different treatment modalities on T‐LGL and how it affects patients' risk of SPMs could also give us further insight into the best management strategies for this patient population.

The primary strength of our study lies in its substantial sample size, given the rarity of the disease. However, several notable limitations, primarily associated with its dependence on a registry, must be acknowledged. Limitations to the use of the registry include the reliability of diagnostic workup at the some cancer centers, without the ability to confirm diagnosis of T‐LGL from reactive T‐cell populations. Given the limitations of the database, data could not be adjusted for existing comorbidities, previous therapies used that may be risk factors for certain malignancies, or autoimmune diseases. Another significant constraint is the absence of detailed information on specific therapeutic exposure and cause of death of the patients. In addition, the retrospective nature of the data introduces inherent limitations, contributing to potential selection bias.

The risk of developing SPMs is elevated in patients with T‐LGL, with a heightened risk of secondary hematological malignancies observed within the first 10 years after diagnosis, while the overall risk of secondary solid tumor malignancies increases after this initial 10‐year period. Our studies may help guide survivorship as close follow‐up is needed for T‐LGL patients even after 10 years of diagnosis. Our findings indicate a need for a more standardized surveillance strategy for individuals diagnosed with T‐LGL. Further studies are warranted to understand the underlying mechanisms, delineate potential confounding effects of therapeutic exposures and coexisting rheumatologic and autoimmune disorders in this population which will help develop risk‐adapted surveillance strategies to prevent SPMs in these patients. However, this study emphasizes the need for close monitoring by hematologists and primary care physicians in survivors.

## Author Contributions

Conceptualization of the study and design: Arya Mariam Roy. Data acquisition, interpretation of data, and statistical analysis: Arya Mariam Roy and Richa Parikh. Initial draft of the manuscript: Arya Mariam Roy and Sawyer Bawek. Review and edits of manuscript: All authors. Supervision: Paola Ghione.

## Ethics Statement

The authors have nothing to report.

## Consent

The authors have nothing to report.

## Conflicts of Interest

The authors declare no conflicts of interest.

## Data Availability

The data that support the findings of this study are openly available at https://seer.cancer.gov/.
